# Timing of Hepatic Artery Reperfusion and Biliary Strictures in Liver Transplantation

**DOI:** 10.1155/2013/757389

**Published:** 2013-12-03

**Authors:** Ganesh Gunasekaran, Jyoti Sharma, Leandro C. Mosna, Roxana Bodin, David C. Wolf

**Affiliations:** Division of Hepatobiliary Surgery and Transplantation, Westchester Medical Center, 100 Woods Road, Valhalla, NY 10595, USA

## Abstract

During orthotopic liver transplantation (OLT), biliary tract perfusion occurs with hepatic artery reperfusion (HARP), commonly performed after the portal vein reperfusion (PVRP). We examined whether the average time interval between PVRP and HARP impacted on postoperative biliary strictures occurrence. Patients undergoing OLT from 2007 to 2009 were included if they were ≥18 years old, had survived 3 months postoperatively, and had data for PVRP and HARP. Patients receiving allografts from DCD donors were excluded. Patients were followed for 6 months post-OLT. Seventy-five patients met the study inclusion criteria. Of these, 10 patients had a biliary stricture. There was no statistical difference between those with and without biliary stricture in age, gender, etiology, MELD score, graft survival, and time interval between PVRP and HARP. Ninety percent of patients with biliary stricture had a PVRP-HARP time interval >30 minutes, as opposed to 77% of patients without biliary stricture. However, this was not statistically significant. The cold ischemia time was significantly different between the two groups. Time interval for HARP after PVRP did not appear to affect the development of biliary strictures. However, 30 minutes may be suggested as a critical time after which there is an increase in biliary stricture occurrence.

## 1. Introduction

Orthotopic liver transplantation (OLT) remains the gold standard for treatment of end-stage liver disease (ESLD) despite advances in medical treatment and management of complications [1]. Surgically, OLT involves hepatectomy followed by implantation. Implantation includes reestablishment of three critical structures: the portal vein, the hepatic artery, and the biliary duct in a sequential fashion. Most often, hepatic artery reperfusion (HARP) occurs after portal vein reperfusion (PVRP) and establishes the blood supply to the bile duct epithelium. Postoperative biliary complications may be attributable to hepatic artery thrombosis or stenosis, technical reasons, ischemia-reperfusion injury, and immunological injury. Most common biliary complications include stricture, leak, biloma, and biliary abscess. These complications can be early, those occurring less than 30 days after OLT, or late, those occurring after 30 days [2].

Biliary complications secondary to long warm ischemia times, independent of vascular compromise, have been reported in the literature specifically in recipients who received livers from donation after cardiac death (DCD) donors [3–6]. Ischemic cholangiopathy has been described in 9–50% of DCD recipients with subsequent increased risk for graft loss, retransplantation, and death [3, 6].

Although the PVRP-HARP interval and its effect on biliary complications have been studied before [7], this study aimed to identify the specific PVRP-HARP time interval that is a “cut off” or safe to perform the arterial anastomosis leading to lower postoperative biliary strictures in OLT. We hypothesized that a longer time interval for HARP may result in increased biliary strictures.

## 2. Methods

We retrospectively reviewed all patients who underwent OLT from January 2007 to December 2009 through the Organ Transplant Tracking Record after study approval from the Institutional Review Board. We obtained information on all patients transplanted including demographics, reasons for transplantation, laboratory values pretransplant, operative reports with reperfusion times, and postoperative records to detect biliary strictures.

Patients were included in the study if they were ≥18 years old, had survived the initial OLT and 3 months postoperatively, and had data for PVRP and HARP. Patients receiving allografts from DCD donors, pediatrics patients, retransplantations, split liver transplants, and cases in which arterial complications occurred before biliary strictures were excluded.

Independent variables of interest included; (1) demographics-age, gender; (2) etiology of ESLD; (3) MELD; (4) presence of hepatocellular carcinoma (HCC); (5) status; and (6) year of OLT. Outcomes of interest were graft survival, average PVRP-HARP time interval, and biliary strictures. Biliary strictures were detected via endoscopic retrograde cholangiopancreatography (ERCP) with intervention (i.e., stent placement, etc.) after they were suspected based on laboratory parameters (increased bilirubin, alkaline phosphatase, and gamma-glutamyltransferase) and/or ultrasound findings (biliary ducts dilatation). Mild bile duct stenoses at the anastamosis detected on ERCP not requiring intervention were excluded. These patients were followed with routine monthly labs and ultrasound to ensure that they were excluded appropriately.

Patients were followed for 6 months after-OLT to see if they developed any biliary strictures. Organ procurement technique was performed in the standard fashion as described by Starzl. Aortic and portal flush (through inferior mesenteric vein cannulation) were used systematically with HTK preservation solution (4 liters for systemic and 2 liters for portal circulation). The biliary tract was flushed with cold saline through the gallbladder after the bile duct was divided. Immunosuppression included calcineurin inhibitor, myfortic, and steroid taper. Rejection episodes were confirmed by liver biopsy and treated with steroid boluses or thymoglobulin based on individual cases.

Biliary reconstruction type was choledochocholedochostomy performed in an end-to-end fashion with 5-0 PDS running suture in all cases. Artery anastomoses were performed with 6-0 or 7-0 Prolene running sutures in an end-to-end fashion between donor and recipient common hepatic arteries. Three different senior surgeons performed the total of the cases assisted by a junior surgeon.

All data were analyzed using SPSS. Statistical analyses were reported as percentage or means with standard deviations. Two groups (no biliary strictures versus biliary strictures) were compared using the Pearson's chi-test, chi-square test, or the Fisher's exact test when appropriate.

## 3. Results

One hundred sixty patients underwent OLT from January 2007 to December 2009. Of these only 75 patients met all inclusion criteria to be included within the study. Ten of the 75 patients had a biliary stricture within 6 months of OLT. There was no statistical significant difference between patients with and without biliary strictures in age, gender, etiology, HCC, mortality status (alive or deceased), year of transplantation as well as the mean MELD, graft survival, and PVRP-HARP time interval. However, patients with biliary strictures did have longer warm (*P* = 0.06) and cold ischemia time (*P* = 0.03) compared to those without biliary strictures (Table 1).

**Figure 1:**
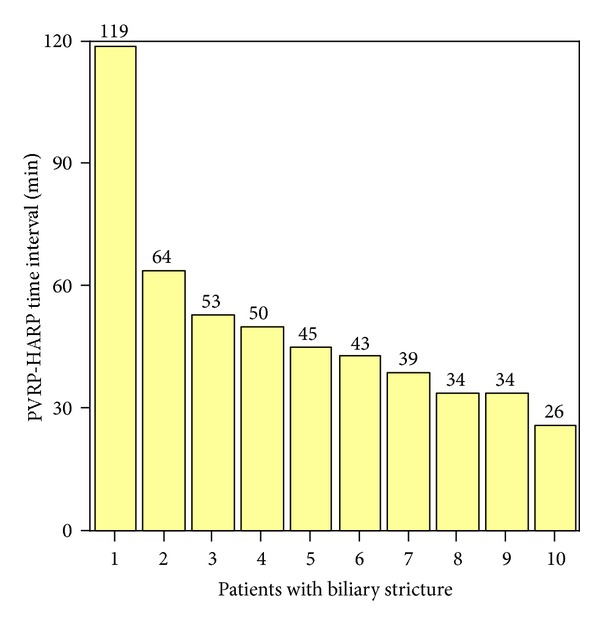
Range of PVRP-HARP time intervals in OLT patients with biliary strictures (*N* = 10) from 2007 to 2009.

The average PVRP-HARP time was 50 minutes for 10 patients with biliary strictures. Figure 1 displays the range of PVRP-HARP in patients with biliary strictures. Most PVRP-HARP time intervals were larger than 30 minutes (only 1 patient had a PVRP-HARP time interval less than 30 minutes) (Figure 1).

Three categories of PVRP-HARP time intervals were compared between patients with and without biliary strictures including 30 minutes, 48 minutes (the average PVRP-HARP time interval of all OLT), and 1 hour (Table 2). Nine of 10 (90%) patients with biliary strictures had a PVRP-HARP time interval >30 minutes, as opposed to 50 of 65 (77%) patients without biliary strictures. However, this was not statistical significantly. The cold ischemia time was significantly different between the two groups (*P* = 0.03).

## 4. Discussion

Warm ischemia time is the duration the liver is removed from ice until it is reperfused with recipient blood. During warm ischemia time, the liver is susceptible to both ischemia and eventually reperfusion injury [8]. Although initial hypothermia halts metabolism, slow depletion of cellular energy stores occurs and rewarming at time of reimplantation causes an increase metabolic demands without oxygen and nutrients, leading to structural and functional cellular defects [8]. Thus, due to this accelerated cellular damage during rewarming, a relatively short period of warm ischemia is more harmful to cells than a longer cold ischemia time [8]. One population in which the effect of warm ischemia has been widely studied is the DCD population.

DCD is the fastest growing source of transplanted livers in the US [3]. The first reports of DCD organ procurement were published in 1995 by University of Pittsburgh [9] and Madison, WI [10]. DCD can be either uncontrolled or controlled [4, 11]. In the former patients have unexpected cardiopulmonary arrest and/or unsuccessful resuscitation, and in the latter they undergo planned withdrawal of ventilatory and organ-perfusion support most often in the operating room [4, 11]. Uncontrolled DCD is associated with a severe ischemic injury resulting in inferior recipient outcomes [11]. Initial enthusiasm for DCD was tempered by the less favorable outcomes compared with DBD. Currently, most centers accept controlled DCD livers with their own selection criteria.

The biliary epithelium is particularly susceptible to the ischemia-perfusion injury mentioned above [12]. Thus, DCD donors have higher incidence of biliary strictures and/or bile cast syndrome [5, 6, 11, 13, 14] as well as ischemic cholangiopathy, which has been reported in 9–50% of DCD recipients [4, 6, 11–16].

With improvements in organ selection, retrieval, preservation, and implantation techniques, biliary complications have dramatically reduced since the 1970s. However, biliary complications still occur 10–30% of OLT, resulting in death for 10% of patients [2, 17–23]. The most common biliary complications are leaks and strictures, but sphincter of Oddi dysfunction, hemobilia, and biliary obstruction from cystic duct mucocele, stones, sludge, or casts are also possible [17, 24, 25]. Similar to other study findings [2], our biliary strictures were primary treated with ERCP and stenting. Postintervention, our patients did well and likely have a long-term success rate of >50–70% [26–31].

This study examined the time interval between PVRP and HARP to determine its effect on postoperative biliary strictures in DBD OLT. We found that the length of the interval between PVRP-HARP did not impact the incidence of biliary strictures up to 6 months after OLT. Moreover, none of our 10 patients with biliary strictures had ischemic cholangiopathy, which has been reported in up to 50% of DCD recipients [4, 6, 11–16]. This may be due to the physiological differences (i.e., longer warm ischemia) in DCD versus DBD organ procurement. Animal models have indicated that dual vessel reperfusion (both vein and artery reperfused at the same time) has higher bile flow and biliary cholesterol than single reperfusion (vein only), leading to better liver function in the former [32]. Our study implies that at least in terms of biliary complication dual vessel reperfusion is not crucial. Also, variation in timing of hepatic artery preparation (i.e., prior to implantation, after implantation and PVRP or a combination) likely does not affect biliary stricture occurrence postoperatively. Interestingly, almost all our patients with a biliary stricture had a PVRP-HARP time above 30 minutes, although not statistically significant this may be clinically significant with 30 minutes being a critical time after which there is an increase in biliary stricture development.

In our study, longer warm and cold ischemia time, especially the latter, were seen in patients with biliary strictures. These findings are consistent with other studies in DCD OLT, where cold and warm ischemia time were risk factors for predicting postoperative biliary complications [33]. We attempted to determine in this study whether other time-sensitive factors (i.e., PVRP-HARP time interval) impact the development of biliary strictures.

There are some limitations to our study. First, of all the possible OLT patients available for this study, 160, only 75 were met the inclusion criteria. A significant number of these patients were excluded because they lacked PVRP and HARP data values, or the values were not recorded correctly. This was particularly true in the first year of our study, 2007. Secondly, our small sample size prevented the study from having enough power to find any statistical significance in our results. Finally, the cause of biliary strictures in our subjects could be multifactorial, and the retrospective nature of this study further prevents us from drawing any temporal conclusions of causality or generalizing our results to other liver transplant populations. Despite these limitations, this study does give us insight with regard to timing of hepatic artery reperfusion and OLT outcomes.

## 5. Conclusion

The length of the time interval for HARP after PVRP did not appear to affect the development of biliary strictures. On the other hand, the cold ischemia time did appear to be significant, in accordance with the previous literature [7, 33]. Although no statistically significant difference in PVRP-HARP time was found between the groups with and without biliary strictures, it is possible to suggest a potential critical time of 30 minutes based on the observation that 9 out of 10 biliary strictures occurred when the PVRP-HARP interval was greater than that time. Larger prospective randomized studies with longer follow-up time are needed to determine if the PVRP-HARP time interval impacts postoperative biliary strictures development.

## Figures and Tables

**Table 1:** Descriptive characteristics of patients with and without biliary strictures undergoing OLT from 2007 to 2009.

Variable	OLT without biliary strictures (*N* = 65)	OLT with biliary strictures (*N* = 10)	*P* value
Age, years, mean ± sd (range)	55.0 ± 11.3 (18.0–75.0)	55.0 ± 9.9 (37.0–70.0)	0.99
Male, % (*N*)	72.3 (47)	80.0 (8)	0.60
Etiology, % (*N*)			
HCC	27.7 (18)	30.0 (3)	0.57
ALF/fulminant	4.6 (3)	10.0 (1)	0.44
HCV/ETOH	4.6 (3)	10.0 (1)	0.44
HCV	21.5 (14)	20.0 (2)	0.63
HBV	3.1 (2)	0.0 (0)	0.75
PSC	3.1 (2)	0.0 (0)	0.75
NASH	3.1 (2)	0.0 (0)	0.75
Cryptogenic	10.8 (7)	20.0 (2)	0.34
MELD, mean ± sd (range)	21.4 ± 10.5 (6.0–44.0)	24.3 ± 12.7 (6.0–48.0)	0.42
Graft survival, months, mean ± sd (range)	24.9 ± 9.5 (3.0–45.0)	24.9 ± 9.6 (12.0–40.0)	0.99
Alive, % (*N*)	86.2 (56)	90.0 (9)	0.74
PVRP-HARP, minutes, mean ± sd (range)	48 ± 29 (10–189)	50 ± 26 (26–119)	0.81
Warm ischemia time (WIT; minutes) mean ± sd (range)	41.3 ± 7.8 (20–60)	46.8 ± 12.2 (36–69)	0.06
Cold ischemia time (CIT; minutes) mean ± sd (range)	407.9 ± 126.6 (38–756)	501.5 ± 125.9 (277–631)	0.03*

*Statistically significant.

**Table 2:** Comparison of various PVRP-HARP time intervals and frequency of biliary strictures in OLT patients (*n* = 75) from 2007 to 2009.

		Biliary strictures			
		NO	YES	Pearson's chi test	*P* value
Time interval between PVRP-HARP (minutes)	<30	15	1	0.88	0.68
	≥30	50	9		
	<48	38	6	<0.000	1.00
	≥48	26	4		
	<60	46	7	0.002	1.00
	≥60	19	3		

## Abbreviations

DBD: Donation after brain death
DCD: Donation after cardiac death
ERCP: Endoscopic retrograde cholangiopancreatography
ESLD: End-stage liver disease
HARP: Hepatic artery reperfusion
OLT: Orthotopic liver transplantation
PVRP: Portal vein reperfusion.
